# Immunological and genomic characterization of Ibizan Hound dogs in an endemic *Leishmania infantum* region

**DOI:** 10.1186/s13071-022-05504-3

**Published:** 2022-11-28

**Authors:** Luis Álvarez, Pablo-Jesús Marín-García, Lola Llobat

**Affiliations:** Departamento Producción y Sanidad Animal, Salud Pública y Ciencia y Tecnología de los Alimentos. Facultad de Veterinaria, Universidad Cardenal Herrera-CEU, CEU Universities, Valencia, Spain

**Keywords:** Cytokines, Genetic regulation, Genomic analysis, Ibizan Hound, Immune response

## Abstract

**Background:**

The Ibizan Hound is a canine breed native to the Mediterranean region, where leishmaniosis is an endemic zoonosis. Several studies indicate low prevalence of this disease in these dogs but the underlying molecular mechanism remains unknown.

**Methods:**

In this study, qualitative immunological and genomic profiles of this breed have been analyzed.

**Results:**

Our analysis shows relevant differences between the cytokine serum profile of Ibizan Hound dogs and previously published data from other canine strains. Additionally, several genetic risk variants related to the immune response, regulation of the immune system, and genes encoding cytokines and their receptors have been studied. The most relevant genes that presented such fixed polymorphisms were *IFNG* and *IL6R.* Other variants with frequencies ≥ 0.7 were found in the genes *ARHGAP18*, *DAPK1*, *GNAI2*, *MITF*, *IL12RB1, LTBP1, SCL28A3*, *SCL35D2, PTPN22, CIITA*, *THEMIS*, and *CD180*. Epigenetic regulatory genes such as *HEY2* and *L3MBTL3* showed also intronic polymorphisms.

**Conclusions:**

Our analysis and results indicate that the regulation of immune responses is different in Ibizan Hounds compared to other breeds. Future studies are needed to elucidate whether these differences are related to the low prevalence of *L. infantum* infection in the Ibizan Hound.

**Graphical Abstract:**

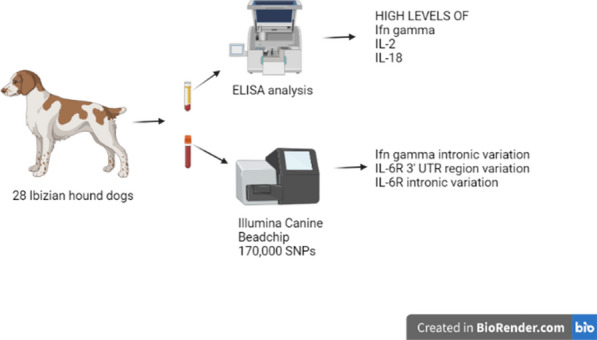

**Supplementary Information:**

The online version contains supplementary material available at 10.1186/s13071-022-05504-3.

## Background

The Ibizan Hound, also named “Podenco Ibicenco” or “Ca Eivissenc,” is the oldest of the 32 Spanish dog breeds officially recognized by the Spanish Kennel Club and the “Federation Cynologique Internationale.” The Federation classified the Ibizan Hound breed with the standard number 89, in group 5-Sect. 7 (Spitz and primitive types-primitive type hunting dogs), being accepted, without working trial in Spain since 1954, with an official valid standard since 1982 and official genealogical book since 2007 [1]. Although the Ibizan Hound breed is native to the Balearic Islands, it is also quite abundant in other Mediterranean regions in Spain such as Catalonia or Valencia, and in France, such as Roussillon or Provence. According to The American Kennel Club, the main physical characteristics of the Ibizan Hound are: (i) height ranging between 23.5 and 27.5 inches in males and 22.5 and 26 inches in females; (ii) weight ranging between 45 and 50 pounds in both males and females; (iii) mean life expectancy ranging between 11 and 14 years in both sexes [1].

Hunting dogs have an increased risk of infectious disease transmission due to (i) direct or indirect contacts with wildlife reservoirs, (ii) exposure to vector insects and ticks, and (iii) feeding habits like entrail ingestion in areas where downed game animals are field dressed [2]. Leishmaniosis is considered one of the most relevant vector-borne diseases in the Mediterranean region, where it is zoonotic, and its seroprevalence is estimated between 63 and 80% of the domestic dog population [3, 4], causing around 20,000–40,000 deaths per year in humans [5]. This disease is caused by different species of the genus *Leishmania*, including *L. infantum*, the most prevalent causal agent of leishmaniosis in the Mediterranean area, which is transmitted by *Phlebotomus* spp. [6]*.*

The global seroprevalence in domestic dogs in Spain is estimated in 10.1%, one of the highest incidedences in the Mediterranean region [7]. In a recent study from Llobat’s group exploring 26 canine breeds, not including Ibizan Hounds, estimated the prevalence of *L. infantum* infection in the Ibizan Islands to be 4.7% (positive animals with or without clinical signs), being the most prevalent in the Doberman Pinscher and Boxer breeds, with seroprevalences of 42.9% and 39.1%, respectively [8].

Several studies have shown differences in immune response against *L. infantum* infection in Ibizan Hounds. Burhnam et al. [9] found higher concentrations of anti-*Phlebotomus perniciosus* antibodies in saliva and concomitant increase of interferon-γ (IFN-γ) serum levels in Ibizan Hounds compared to other canine breeds. Therefore, it has been proposed that such high IFN-γ values have a protective effect, since they induce the cellular immune response [10]. Moreover, Solano-Gallego et al. (2000) demonstrated a different specific immune response against *L. infantum* in Ibizan Hound specimens compared to other canine breeds, showing that 81% of Ibizan Hound dogs analyzed presented a cellular immune response vs. only 48% presented in the other dogs [11].

In addition, some genomic analyses suggest that several polymorphisms are involved in the molecular mechanisms associated with the resistance or susceptibility to canine leishmaniosis. Two polymorphisms present in the canine *Slc11a1* gene, one intronic single-nucleotide polymorphism (SNP) and one silent SNP in exon 8, have been associated with an increased risk of canine visceral leishmaniosis [12]. Four SNPs in the canine β-defensin-1 (*CBD1*) gene have been associated with *L. infantum* infection in dogs from Italy [13].

As far as we know, no genomic studies in Ibizan Hound have been perfomed focusing on the immune response against *L. infantum*. Here, we present an immunological and genetic characterization of the Ibizan Hound breed to shed light on the underlying mechanisms leading to different immune responses.

## Methods

### Animal and epidemiological data

Twenty-eight Ibizan Hounds were analyzed. For all animals, epidemiological data were collected: sex, age, general and leishmaniosis vaccination status, external deworming status and type, living conditions (indoors or outdoors), type of parasitic feeding burden, and clinical signs.

### Sample collection and cytokine levels

Ten milliliters of whole blood was taken by cephalic venipuncture using Vacutainer tubes without anticoagulant. Samples were maintained at room temperature to obtain serum aliquots, which were stored at − 20˚C until processing. Serological testing for *L. infantum* detection of specific antibodies was performed using the indirect immunofluorescent antibody test (IFAT) for anti-*Leishmania*-specific immunoglobulin G (IgG) antibodies (MegaFLUO^®^ LEISH, Megacor Diagnostik GmbH, Hörbranz, Austria). Samples were considered seropositive with IFAT titer ≥ 1/80, following the manufacturer’s instructions. The whole blood samples were used for DNA extraction before 24 h to recovery.

The IL-2, IL-6, IL-8, IFN-γ (Canine IL-2 ELISA kit, Canine IL-6 ELISA kit, Canine IL-8 ELISA kit, and Canine IFN-γ ELISA kit, respectively; Invitrogen, Waltham, MA, USA,), and IL-18 (Canine IL-18 ELISA kit, Mybiosource, San Diego, CA, USA) levels were measured in serum samples by commercial ELISA kit method following the manufacturers' recommendations. In brief, 50 µl serum was used for the sandwich ELISA analysis. The microplate had been pre-coated with an antibody specific to cytokines. The samples were added to the microplate wells and combined with the specific antibody. Then, a biotinylated detection antibody specific for each cytokine and avidin-horseradish peroxidase (HRP) conjugate were added successively to each microplate well and incubated. Free components were washed away. The substrate solution was added to each well. The enzyme-substrate reaction was determined by the optical density (OD) and measured spectrophotometrically at a 450-nm wavelength in a Victor-X3™ plate reader (Perkin Elmer^®^). The concentration of each cytokine was calculated by comparing the OD of the samples to the standard curve.

### DNA extraction and whole genome analysis

Genomic DNA (gDNA) from 28 Ibizan Hound samples was isolated using a QIAamp DNA Blood Kit following the manufacturer’s protocol (QIAamp; Qiagen, Hilden, Germany). DNA was quantified using a Nanodrop spectrophotometer (Thermo Fisher Scientific), and only samples with A_260_/A_280_ ratio > 1.8 were used. gDNA concentrations for all samples were a minimum of 50 ng/µl. DNA samples were whole genome amplified for 20–24 h at 37 ºC, fragmented, precipitated and resuspended in an appropriate hybridization buffer.

Samples were genotyped using the CanineHD DNA Analysis BeadChip WG-440–1001 (Illumina, Inc., San Diego, CA, USA) and hybridized on the prepared BeadChips for 16–24 h at 48 ºC. Following the hybridization, nonspecifically hybridized samples were removed by washing, while the remaining specifically hybridized loci were processed for the single-base extension reaction, stained, and imaged on an Illumina iScan Reader. GenomeStudio 2.0.5 (Illumina Inc., San Diego, CA, USA) was used to process data generated from the iScan system for subsequent analysis, according to manufacturer guidelines. Intensity data were loaded into the Genotyping Module to primary data analysis, including raw data normalization, clustering, and genotype calling.

SNPs on sexual chromosomes and with a call rate < 95% were discarded using PLINK v1.90b6.22. The final data set included 165,480 mapped positions in 24 Ibizan Hound samples with a mean genotyping rate of 0.988.

### Analysis of polymorphisms related to immune response

The allelic status of previously published polymorphisms and genomic regions described as related to the immune response to *L. infantum* infection in dogs [12, 14–19] were interrogated in the analyzed samples.

PLINK v1.90b6.22 was used to extract variants from the selected genome regions, encompassing 38 genes, according to the mapping information of the *Canis lupus familiaris* genome assembly CanFam3.1. and to calculate allele frequencies. Polymorphisms were considered fixed in the Ibizan Hound breed when presenting frequencies > 0.7. Detected polymorphisms were annotated using NCBI refseqs, release 105. The rsID information was downloaded and annotated from the European Variation Archive EVA release 3 files corresponding to the CanFam3.1 assembly.

## Results

First, we studied relevant epidemiological parameters of the Ibizan Hound such as gender, age, and diet; 60.71% of the analyzed Ibizan Hound specimens were males, categorized as adults (32.14%) or elderly (39.29%). Most (82.14%) were fed commercial food (Table 1). According to their lifestyle, all animals lived outdoors in contact with other dogs, used external deworming, and had the required vaccinations. None of the subjects were vaccinated against *Leishmania*. Likewise, none of the animals included in this study presented a positive result for antibodies against *Leishmania* according to the IFAT technique. Serum levels of the analyzed cytokines are summarized in Table 2.

**Table 1:** Epidemiological data of the 28 Ibizan Hound samples analyzed

Variable	Categories	No. of dogs (%)
Gender	Male	17 (60.71)
	Female	11 (39.29)
Age	Puppy (< 1 year)	2 (7.14)
	Young (1 to 5 years)	6 (21.43)
	Adult (5 to 10 years)	9 (32.14)
	Elder (> 10 years)	11 (39.29)
Diet	Commercial	23 (82.14)
	Home prepared/raw food consumption	5 (17.86)
Overall		28 (100.00)

**Table 2:** Concentration values of the analyzed cytokines

Cytokine^1^	n	Range^2^	Mean ± SD^2^	CV (%)
IFN-γ	28	0.05–3.06	0.65 ± 0.30	46.15
IL-2	28	26.52–97.10	64.55 ± 18.62	28.85
IL-6	28	0.41–0.99	0.62 ± 0.12	19.35
IL-8	27	14.09–619.05	160.55 ± 124.50	77.55
IL-18	27	100.95–1514.45	529.97 ± 293.97	55.47

The table shows the number of animals (n), range, mean ± standard deviation (SD), and coefficient of variation (CV)

^1^IFN-γ: interferon gamma, IL: interleukin

^2^The values for IL-8 are expressed pg/ml and for IFN-γ, IL-2, IL-6, and IL-18 are expressed as ng/ml

Second, we investigated the incidence of genomic variance in our cohort. In Tables 3, 4 and Additional file 1, information on 217 polymorphisms has been analyzed in 38 immune response-related genes. Table 3 summarizes the cytokine genes variants found in the genomic positions and their frequencies in the analyzed samples. In the IFN-γ gene, which is encoded by a gene located at chromosome 10, one polymorphism (rs22078594) was found with a frequency of 0.86 of the alternative alleles (A). In the *IL2* gene, located at chromosome 2, a different variant was identified (rs22707631) in 3’ UTR with a 0.63 frequency of the alternative allele. No variants were found in the *IL6* gene in chromosome 14; however, the gene that encodes its receptor, located at chromosome 7, showed two variants. The first variant rs24423644 in the 19:17,756,202 position was the most relevant (frequency of the alternative allele = 1) and the second rs24416159 in 7:42,902,063 position (frequency of the alternative allele = 0.78).

**Table 3:** Genomic variants found in genes encoding analyzed cytokines and their receptors

Gene encodes cytokine, receptor, or gene related^1^	rsID	Chromosome position	Ref.–alt.^2^	Frequency	Functional class of variant
IFN-γ (*IFNG*)	rs22078594	10:10,408,724	G-A	**0.86**	Intronic
IL-2 (*IL2*)	rs22707631	19: 17,756,202	T-C	0.63	3′UTR
IL-6 receptor (*IL6R*)	rs24423644	7: 42,878,605	C-T	**1.00**	3′UTR
	rs24416159	7: 42,902,063	G-A	**0.78**	Intronic
	rs24433326	7: 42,888,754	T-C	0.22	Intronic
IL-6 cytokine family signal transducer (*IL6ST*) gene	rs22868191	2:43,260,700	T-C	0.15	Intronic

Table shows the variant identification (rsID), chromosome position, reference and alternative alleles, frequency of alternative variant in the analyzed samples (those with frequencies > 0.7 are highlighted in bold) and functional class of variant

^1^IFN- γ: interferon gamma, IL: interleukin

^2^C: cytosine, T: thymine; A: adenine; G: guanine

**Table 4:** Genomic variants found in genes other than cytokines analyzed with frequency of the alternative allele > 0.7

Gene^1^	rsID	Chromosome position	Ref.-alt.^2^	Frequency	Functional class of variant
*AEED1*	rs21889091	1: 70,201,861	G-A	1	Intronic
*ARHGAP18*	rs21879501	1: 68,152,162	G-A	1	Intronic
	rs22026852	1: 68,221,142	T-C	0.96	Intronic
	rs21954955	1: 68,225,797	T-C	0.93	Intronic
	rs9165143	1: 68,178,311	C-T	0.76	Intronic
	rs21905342	1: 68,254,443	T-C	0.71	Intronic
	rs21948539	1: 68,258,523	T-C	0.70	Intronic
*CD180*	rs22872722	2:52,660,671	T-C	0.77	5′UTR
*CIITA*	rs24353887	6:31,796,528	A-G	1.00	Intronic
	rs24328818	6:31,803,906	T-C	0.96	Intronic
*CLVS2*	rs21898983	1: 62,276,253	C-T	0.80	Intronic
*DAPK1*	rs21900944	1: 72,367,869	G-A	0.78	Intronic
*ECHDC1*	rs21938035	1: 66,013,856	A-G	0.87	Intronic
*GNAI2*	rs22932270	20:39,083,622	G-C	0.96	Intronic
*GOLM1*	rs21950391	1: 73,631,884	T-C	0.76	Intronic
*HABP4*	rs21886036	1: 70,370,888	T-A	0.91	Intronic
*HEY2*	rs9207199	1: 64,638,097	C-A	0.91	Intronic
*IL12RB1*	rs22916906	20:44,890,151	C-T	0.78	Intronic
	rs22844706	20:44,906,998	G-C	0.78	Downstream
*L3MBTL3*	rs21966134	1: 68,652,597	T-C	0.83	Intronic
*LAMA2*	rs22024503	1: 7,706,395	A-G	1	Intronic
	rs22040055	1: 67,843,651	A-C	1	Intronic
	rs21975303	1: 68,074,486	T-C	1	Intronic
	rs851817104	1: 67,689,922	A-G	0.98	Intronic
	rs21912280	1: 67,694,283	T-C	0.98	Synonymous
	rs21909819	1: 67,718,504	T-C	0.92	Intronic
	rs21978490	1: 67,728,530	A-G	0.92	Intronic
	rs21901417	1: 67,922,585	G-A	0.73	Intronic
*LTBP1*	rs22600228	17:26,509,918	C-T	1.00	Intronic
	rs22585937	17:26,469,383	T-C	1.00	Intronic
	rs22565021	17:26,402,720	C-T	1.00	Intronic
	rs22617477	17:26,375,163	G-A	1.00	Intronic
	rs22564606	17:26,144,284	C-T	0.98	Intronic
	rs22598552	17:26,233,826	G-T	0.94	Intronic
	rs22583693	17:26,283,669	G-A	0.92	Intronic
	rs22600112	17:26,478,030	G-A	0.92	Intronic
	rs22585869	17:26,451,018	C-G	0.87	Intronic
	rs22564554	17:26,133,100	G-A	0.83	Intronic
	rs22584546	17:26,513,340	T-C	0.79	Intronic
	rs22569416	17:26,520,738	A-G	0.79	Intronic
	rs22600186	17:26,500,115	C-T	0.73	Intronic
*MITF*	rs22864511	20:21,848,178	A-C	1.00	Intronic
*PKIB*	rs21960517	1: 62,012,877	G-A	0.85	Intronic
*PTPN22*	rs22559491	17:51,651,794	A-T	1.00	Intronic
	rs22559538	17:51,663,769	G-A	1.00	Intronic
	rs22582077	17:51,667,891	C-T	1.00	Intronic
	rs22559449	17:51,633,544	A-G	0.90	Intronic
*PTPRK*	rs21887741	1: 66,895,650	G-C	1	Intronic
	rs8721457	1: 66,692,393	G-C	0.98	Intronic
	rs22013934	1: 66,866,914	T-C	0.98	Intronic
	rs21951775	1: 66,740,449	A-G	0.98	Intronic
	rs21939204	1: 66,833,054	T-C	0.98	Intronic
	rs21951655	1: 66,940,821	G-T	0.80	Intronic
	rs22038850	1: 66,626,458	G-T	0.78	Intronic
	rs21963966	1: 66,635,731	C-T	0.78	Intronic
	rs21890648	1: 66,648,882	A-G	0.77	Intronic
	rs21914202	1: 66,663,772	C-T	0.77	Intronic
	rs8868883	1: 66,673,590	T-C	0.77	Intronic
	rs21893059	1: 66,612,866	C-T	0.76	Intronic
	rs9021755	1: 6,599,857	C-T	0.75	Intronic
*RAB38*	rs22921195	21:12,120,865	T-C	1.00	Intronic
	rs22917843	21:11,811,693	A-G	1.00	Intronic
	rs22976236	21:11,791,901	G-A	0.96	Intronic
*RASEF*	rs21892604	1: 76,327,161	G-A	1.00	Intergenic
	rs21894538	1: 76,416,494	C-T	1.00	Intronic
	rs21913661	1: 76,423,140	A-G	1.00	Intronic
	rs21984010	1: 76,452,566	G-A	1.00	Missense
	rs21965379	1: 76,432,736	C-T	0.98	Intronic
	rs21964231	1: 76,456,202	T-C	0.96	Intronic
	rs21969829	1: 76,395,909	C-T	0.92	Intronic
*RSPO3*	rs21964628	1: 65,935,687	G-C	1.00	Intronic
	rs8881600	1: 65,858,997	C-T	0.93	Intronic
	rs9173069	1: 65,864,484	T-C	0.85	Intronic
	rs22026455	1: 65,912,368	G-T	0.80	Intronic
	rs21975949	1: 65,900,327	C-T	0.77	Intronic
	rs9255550	1: 65,884,205	A-C	0.76	Intronic
	rs21978415	1: 65,928,148	G-A	0.76	Intronic
*SLC28A3*	rs9157044	1: 75,042,990	C-A	0.90	Intronic
*SLC35D2*	rs21998807	1: 70,501,388	A-G	0.94	Intronic
*SERINC1*	rs21937411	1: 61,830,421	G-T	1.00	Missense
*THEMIS*	rs21975824	1: 66,376,836	T-C	0.98	Intronic
	rs9237291	1: 66,433,882	C-T	0.98	Intronic
	rs9158578	1: 6,416,081	A-C	0.95	3'UTR
	rs8659520	1: 66,516,280	A-G	0.95	Intronic
	rs21918658	1: 66,399,264	A-G	0.73	Intergenic
*TF*	rs23149486	23:30,383,100	A-T	1.00	Intronic

Table shows the identify number (rsID), chromosome position, reference and alternative alleles, frequency of alternative variant in the analyzed samples, and functional class of variant

^1^*AEED1* (or *PRXL2C*): AhpC/TSA antioxidant enzyme domain containing 1 (or Peroxiredoxin like 2C); *ARHGAP18:* Rho GTPase Activating Protein 18; *CIITA:* Class II Major Histocompatibility Complex Activator; *CLVS2*: Clavesin; *DAPK1*: Death-Associated Protein Kinase I; *ECHDC1*: Enoyl-CoA Hydratase Domain-Containing Protein 1; *GNAI2*: G Protein Subunit Alpha I2; *GOLM1*: Golgi Membrane Protein 1; *HABP4*: Hyaluron Binding Protein 4; *HEY2*: Hes Related Family BHLH Transcription Factor with YRPW Motif 2; *IL12RB1:* Interleukin 12 receptor subunit Beta 1; *L3MBTL3*: L3MBTL Histone Methyl-Lysine Binding Protein 3; *LAMA2*: Laminin Subunit Alpha 2; *LTBP1*: Latent Transforming Growth Factor Beta Binding Protein 1; *MITF*: Melanocyte Inducing Transcription Factor; *PKIB*: CAMP-Dependent Protein Kinase Inhibitor Beta; *PTPN22*: Protein Tyrosine Phosphatase Non-Receptor Type 22; *PTPRK*: Protein Tyrosine Phosphatase Receptor Type K; *RAB38*: Member RAS Oncogene Family; *RASEF*: RAS and EF-Hand Domain Containing; *RSPO3*: R-Spondin 3; *SLC28A3*: Solute Carrier Family 28 Member 3; *SLC35D2*: Solute Carrier Family 35 Member D2; *SERINC1*: Serine Incorporator 1; *THEMIS*: Thymocyte Selection Associated; *TF*: transferrin

^2^C: cytosine, T: thymine; A: adenine; G: guanine

Polymorphisms in genes other than cytokines were also found in the analyzed samples. More precisely, intronic variants have been found in Rho GTPase Activating Protein 18 (*ARHGAP18*), Death Associated Protein Kinase 1 (*DAPK1*), G Protein subunit Alpha 2 (*GNAI2*), and Melanocyte Inducing Transcription Factor (*MITF*) genes, some of them with alterative allele frequency of 1. Other intronic and downstream polymorphisms have been found in different genes which encode for other interleukin receptors (*IL12RB1*), interleukin regulators such as Latent Transforming Growth Factor-β binding protein (*LTBP1*), two members of Solute Carrier Family (*SCL28A3* and *SCL35D2*), and Protein Tyrosine Phosphatase Non-Receptor Type 22 (*PTPN22*), several genes related to expression gene regulation by epigenetic mechanisms (*HEY2*, *L3MBTL3*, and *HABP4)*, membrane transport (*ECHDC1* and *RASEF*), and immune system regulators (*CIITA*, *THEMIS*, and *CD180*), among others (Table 4). The results presented in Table 4 show those fixed variants in the population studied. The remaining SNPs were found with lower frequencies (additional file 1: Table S1).

## Discussion

Our results show the immunological and genomic profile of the Ibizan Hound, which is the oldest of the 32 Spanish dog breeds officially recognized by the Spanish Kennel Club. In our analysis, several cytokine levels were compared to those previously published indicating a differential immunological profile. For the first time, serum levels of IL-2, IL-8, and IL-18 are studied in this canine breed.

Previous studies have indicated that the Ibizan Hound has a natural genetic resistance against leishmaniosis due to the early activation of the cellular immune response related to specific cytokines [11, 17, 20]. In fact, our results showed high levels of IFN-γ, IL-2, and IL-18, whereas the serum levels of IL-8 in this breed were lower than in other breeds [21]. The activation of macrophages by IFN-γ has been associated with effective T helper 1 (Th1) cellular immunity and the elimination of intracellular amastigotes and the control of the disease [11]. In Ibizan Hound dogs [9] a higher expression level of IFN-γ was observed in animals positive to the leishmanin skin test (LST) than in dogs with healthy skin in the nonendemic area [22].

Moreover, the upregulation of IFN-γ has been correlated with low disease severity [23]. Furthermore, stimulation with *Leishmania infantum* soluble antigen (LSA) in Ibizan Hound dogs provokes higher IFN-γ serum levels than in other healthy or sick dog breed [24]. The *IFNG* gene is located at chromosome 10 and presents four exons that codify a unique protein with 166 amino acids. Genomic analyses of *IFNG* show a fixed variant 10:10408724G > A (rs22078594) in an intronic region. This polymorphism produces a transcript named *IFNG-201* with no associated biological functions [25]. However, since another intronic variant in *IFNG* has been correlated with both low levels of IFN-γ in humans and susceptibility to cutaneous leishmaniasis [26], more studies on the rs22078594 variant will be needed to elucidate its potential role in the regulation of the immune response.

The host ability to control *Leishmania* infection requires a strong cellular immune response, which is associated with the activation of T helper (Th)-1 cells producing IFN-γ and IL-2 [27, 28]. Additional evidence suggest that both factors have a key protective role in response to *Leishmania* infection [22, 29, 30]. Aslan et al. [34] found that IL-2 expression was negatively correlated with splenic parasite loads in experimentally infected dogs [31]. Our results are in agreement with previous data, indicating a natural resistance against leishmaniosis in Ibizan Hounds compared to other canine breeds [11, 24, 32]. The gene encoding for IL-2 is located at chromosome 19 and presents four exons. The variant found in *IL2* gene 19, 17756202 T > C (rs22707631), overlaps one transcript of this gene [33]. This polymorphism is in the 3'-UTR region of the gene, which could have consequences in its post-transcriptional gene expression. In humans, several polymorphisms have been found in IL2, but it does not seem to predict susceptibility or protection against the development of disease to *Leishmania guyanensis* [34]. The biological changes associated with this variant are not reported in dogs or humans; thus, further studies will be necessary to shed light on the relationship between this 3'-UTR polymorphism and differences in serum levels of cytokines.

Additionally, IL-18 is a cytokine that plays an important role in both types of immunity, innate and acquired, and mediates Th1 response by inducing IFN-γ production in T cells and natural killer cells [35, 36]. Kumar et al. [40] found a significant relationship between susceptibility to visceral leishmaniasis in humans and the cytokine serum levels [37]. In dogs, it has been reported that IL-18 has no prominent role in the infection outcome of the disease [31, 38–41]. There is no evidence about the expression levels of IL-18 in different canine breeds, but similarly to IFN-g and IL-2, it is possible that IL-18 plays an important role in the natural resistance against leishmaniosis in Ibizan Hound dogs. Previous results from our group showed higher levels of IL-18 in Ibizan Hound than in the Boxer breed (data unpublished), which would be in agreement with these studies supporting the theory that the Ibizan Hound has a higher immune response against *Leishmania* infection than other canine breeds. *IL18* gene is located at chromosome 5 and has seven exons. The array used for this study does not include any position in this gene, but it would be interesting to further analyze this gene and its genomic alterations to dissect whether they could regulate the elevated serum levels.

Low IL-8 serum level values were found in Ibizan Hound dogs. These results are consistent with the scarce studies focused on the role of IL-8 in *Leishmania* infection. Notably, IL-8 is an important cytokine for the activation of immune cells, especially neutrophils. Wardini et al. [45] found lower basal levels of IL-8 production in neutrophils in healthy dogs compared with infected animals [42]. A decrease in IL-8 levels has been reported in dogs infected both naturally and experimentally [43]. However, contrarily, it has been stated that amastigotes accumulate and survive in neutrophils in animals with high IL-8 levels [44], although these results are not conclusive. In humans, IL-8 polymorphism has been considered a risk factor for the development of visceral leishmaniasis [45].

Levels of the inflammatory cytokine IL-6 were similar to previously published data for other canine breeds [21]. Several authors found a higher expression of IL-6 in healthy than in experimentally infected sick dogs [20, 39]. Abbehusen et al. [46] demonstrated that serum levels of this cytokine increased in animals after natural and experimental infection [43]. De Lima et al. [49] pointed out that this cytokine is a good marker in active disease [46]. Other authors did not find a relationship between these cytokine levels and the development of leishmaniosis [31, 38]. The *IL6* gene is located at chromosome 14 and presents five exons. No variants of this gene are included in the array. However, several SNPs have been found in the coding region of the IL-6 receptor (*IL6R*) and IL-6 cytokine family signal transducer (*IL6ST*) gene. For example, one SNP in 3'-UTR and one intronic polymorphism of *IL6R* are located at chromosome 7 and are fixed in the Ibizan Hound population studied. The 3’UTR variant results in a transcript named *IL6R-201*, which encodes a protein with 468 amino acids. The intronic polymorphism overlaps the same transcript as 3’UTR. Although these two SNPs are fixed in the Ibizan Hound population, their respective biological functions are largely unknown. Given that the results obtained in the serum levels of this interleukin were similar to those found in other canine breeds [21], it is possible that these variants do not influence them. IL-6 production increases in blood from dogs living in endemic areas of canine leishmaniosis cultured with several Toll-like receptors (TLR), in particular with TLR-4a and TLR-7a, but not with TLR-3a, whereas the IFN-γ production is not affected [47]. Other TLRs are related to *L. infantum* infection, so TLR-2 expression increases in mononucleate cells of the dermis with LST in Ibizan Hound dogs [48]. In human macrophages, the stimulation with agonist of TLR-2 increases the IFN-γ production through the PI3K-mTOR pathway [49]. In fact, in vitro studies have shown the ability of TLR-2 to reduce anti-leishmanial response mediated by IFN-γ [50], and TLR-2 is expressed in inflammatory cells such as biopsy samples of clinically affected skin [48]. The pathways of TLR and cytokines, and the relationship between them and their expression in macrophages of infected and non-infected dogs, and the polymorphisms of genes encoding these TLRs could be studied in future experiments to elucidate the mechanisms of resistance to *L. infantum* infection.

Different polymorphisms of several genes are fixed in the studied cohort of Ibizan Hound. Some are related to immune regulation. More precisely, six intronic SNPs with unknown biological function have been found in *ARHGAP18* gene. This gene is related to regulation of lymphocyte-specific transcription factor interferon regulatory factor 4 (IRF4) [51]. Recently, Sundararaj and Casarotto [54] published a review indicating different functions of IRF4 in immune regulation, including B cell development [52]. Seven intronic variants are fixed in *LAMA2* gene. Although *LAMA2* is mainly related to muscular diseases (see editorial 70), some authors have indicated functions in immune response. For example, *Lama2*-deficient mouse model shows elevated expression of proinflammatory cytokines, concretely, TNF-α and IL1β [53], and Li et al. [57] demonstrated that LAMA2 is a coreceptor to activate T cells upon infection by *S. aureus* [54]. Thirteen intronic polymorphisms have been found in *LTBP1* gene, a known gene involved in T regulatory lymphocyte differentiation [14]. In fact, Batista et al. [14] performed a genome-wide association study of cell-mediate response in dogs infected by *L. infantum*, and their results showed several genetic markers, which explain the phenotypic variance in cytokines expression [14]. These genetic markers are in some genes where fixed variants have been identified in this study, For example, in *LTBP1* and in *IL12RB1*, which is associated with TNF-α and involved in cytokine and chemokine signaling, with one intronic and downstream polymorphism fixed in our dog population. Another SNP fixed in the Ibizan Hound population and related to immune response is *DAPK1* gene variant. DAPK1 protein is related to mTORC1 activation and CD8 + cell function, by a recently discovered mechanism and is necessary to antiviral activity [55, 56] and most likely antiparasitic immune response. Additionally, there are polymorphisms in known genes that regulate the immune response through other pathways, e.g. *GNAI2*, which regulates B cell motility and entrance into lymph nodes [57, 58], *MITF*, inhibitor of IL-1, IL-6, CCL-2, and TNF-α [59], *PTPN22*, a negative regulator of T cell receptor and B cell receptor signaling [60, 61], *CIITA*, a regulator of Il-6 and HLA-II expression [62, 63], and *THEMIS*, which encodes a protein necessary to maintenance of CD8 + T cell response and regulation of cytokine signal [64]. Two family members of Solute Carrier Family (*SLC28A3* and *SLC35D2*) show intronic variants. Polymorphisms in other members of this family have been associated with susceptibility or resistance against visceral leishmaniosis in different dog populations [12, 65–68], probably by regulation of survival in host macrophages to parasite [69], which could be performed by other members of the same family. Finally, genes encoding regulatory proteins involved in epigenetic regulation also show fixed polymorphisms, such as *HEY2*, *L3MBTL3*, and *HABP4. HEY2* is targeted by Notch1 in humans [70] and is involved in immune response activation [71]. Moreover, *HEY2* expression is regulated by the same epigenetic pathway as *NR4A1* [72], a key mediator of T cell dysfunction [73]. Notch signaling is also regulated by *L3MBTL3* as epigenetic regulator [74]. Overall, our results indicate that Ibizan Hound is a canine breed with a singular immune response against *L. infantum* infection and probably against other pathogens. Therefore, further studies will be necessary to elucidate the molecular mechanism underlaying the immune response and its relationship with the low prevalence of leishmaniosis in this breed.

## Conclusions

Several studies indicate that the Ibizan Hound seems to present resistance against *L. infantum* infection, with lower prevalence of leishmaniosis than other canine breeds. This qualitative study shows high levels of IFN-γ, IL-2, and IL-18 in Ibizan Hound dogs, whereas the serum levels of IL-8 in this breed are lower than in other breeds. The genomic analysis reveals many variants which are fixed in this dog population and related to expression and regulation of the immune system. These results indicate that this breed presents a specific immune response, which could explain this low prevalence of leishmaniosis and would be related to resistance against other infection pathogens. Future studies will be necessary to discover the specific biological function of the genetic SNPs found here, more precisely in genes related to expression and regulation of cytokines levels.

## Supplementary Information

**Additional file 1: Table S1.** Genomic variants found in non-cytokine genes with frequency of alternative allele minor than 0.7. Table shows the identify number (rsID), chromosome position, reference and alternative alleles, frequency of alternative variant in the analyzed dataset, and functional class of variant.

## Data Availability

The datasets used and/or analyzed during the current study are available from the corresponding author on reasonable request.
